# Assessment of refining efficiency during the refining cycle in a foundry degassing unit in industrial conditions

**DOI:** 10.1038/s41598-024-51914-x

**Published:** 2024-01-16

**Authors:** Ladislav Socha, Tomáš Prášil, Karel Gryc, Jana Svizelova, Mariola Saternus, Tomasz Merder, Jacek Pieprzyca, Petr Nuska

**Affiliations:** 1 Environmental Research Department, Institute of Technology and Business in České Budějovice, Okružní 517/10, 370 04 České Budějovice, Czech Republic; 2 University of West Bohemia, Univerzitní, 2732, 301 00 Plzeň, Czech Republic; 3 Die-Casting Division, MOTOR JIKOV Slévárna a.S, Kněžskodvorská, 2277, 370 04 České Budějovice, Czech Republic; 4 Faculty of Materials Engineering, Silesian University of Technology, Krasinskiego 8, 40-019 Katowice, Poland

**Keywords:** Design, synthesis and processing, Metals and alloys

## Abstract

The article focuses on the issue of improving the efficiency of a Foundry Degassing Unit (FDU) via operational testing of aluminium alloys during casting at MOTOR JIKOV Slévárna a.s.. As part of the research, the efficiency of the refining process in the FDU was assessed. The main emphasis was placed on determining the moment of the greatest decrease in the hydrogen content in the melt and whether it is possible to shorten the refining cycle. The values of the Dichte Index were determined, on the basis of which the degassing curve was plotted and the progress of the melt degassing was assessed. To ensure the required quality of castings, the maximum allowable value of the Dichte Index ranged from 3 to 4%. During the process, the temperature drop during the refining cycle was also determined. The total temperature drop from pouring the melt into the ladle to the end of refining ranged from 26 to 32 °C, which is within the acceptable limits of the foundry. Based on the knowledge resulting from the operational experiments, recommendations were formulated to optimize the refining technology at the FDU for the MOTOR JIKOV Slévárna a.s. foundry.

## Introduction

As a pure metal, aluminium has low tensile strength and hardness, so it must be alloyed to overcome mechanical strength limits^1^. The properties of the aluminium alloys obtained in this way, especially the high strength-to-weight ratio, resistance to fatigue, impact strength and corrosion, make them some of the most important materials in industrial design^2^. Therefore, aluminium alloys are applied widely in the automotive and aerospace industries, architecture, electronics, hydraulics, semiconductor devices and outdoor recreational products^3^. Aluminium alloys, despite their favourable properties, suffer from the formation of inclusions, porosity, surface undulations, etc.^4^. During alloy processing and casting, these undesirable defects reduce the final quality of the products^5^. It is known that aluminium tends to absorb hydrogen gas, which is a major contributor to the formation of microporosity in aluminium alloys^6^. Aluminium is relatively susceptible to reacting with oxygen during melting, leading to the formation of oxide inclusions^7^. Also, the presence of non-metallic inclusions with poor wettability in molten aluminium is highly undesirable^8^, since they are suspended in the alloy and serve as pore nucleation sites^9^.

For these reasons, refining is an essential technological step in the production of aluminium, and its main purpose is to reduce the content of hydrogen, inclusions and other impurities to an acceptable level in order to improve the overall quality of the alloy and increase the quality of castings^10^. Of the several methods used to remove impurities from liquid aluminium alloys (e.g., flotation, fluxing and filtration), refining using a chemically inactive gas bubbles is the most popular^11^. In recent years, there has been a continuous development of techniques for introducing refining gas to produce high-quality aluminium^12^. This started with nozzles (e.g. reactors: FILD^13^, Jetcleaner, MINT^14^), porous plugs (e.g. reactors: Alcoa 469, DMC, DUFI, URC-7000)^15^ and has now reached a point using rotors with graphite heads (e.g. rotors: ACD^16^, AFD, Alpur, GBF, HYCAST^17^, RDU, SNIF, URO-200). Currently, the refining process is very often carried out in a degassing unit with rotors, known as a foundry degassing unit or FDU^18^. In this method, a chemically inactive gas, such as argon or nitrogen, is introduced through a rotating shaft and impeller^19^. When the rotor rotates at high speed, the bubbles are broken up by the shear force acting on the surface of the rotor^20^. In highly stirred reactors, the behaviour and mechanism of gas bubbles as well as the hydrodynamic field are complicated^21^. The hydrodynamic field is characterized by turbulence and an uneven rate of energy dissipation and the presence of vortices moving in different directions^22^. This means that the bubbles in such systems are exposed to various internal and external forces that lead to their rupture^23^. The rotary speed, mixing force, surface tension and liquid density have the greatest influence on the size of gas bubbles^24^. In fact, the large surface area leads to the efficient and rapid diffusion of hydrogen into the gas bubbles, resulting in the establishment of a balance of hydrogen in the liquid and gas phases, and consequently the complete removal of hydrogen without the use of harmful salts containing chlorine and fluorine^25^. In addition, the presence of small bubbles helps to remove non-metallic solids by flotation^17^. This process is greatly aided by the turbulent flow in the melt, which promotes mass transport and also has a significant impact on flotation efficiency^26^.

The need to improve the quality of products demands a better understanding of the events that occur during the production process^27^. The progress of metallurgical processes can be studied in various ways, and one of them is modelling, which imitates the behaviour of a real system. Given the specified requirements, the behaviour of the real system can be predicted by the model when various conditions change^28^. This method allows phenomena to be studied that are difficult to measure in operating conditions^29^. Since the surrounding medium in the model is more favourable than under operating conditions (e.g. temperature, melt spatter), more sensors and other measuring devices can be used^30^. The topic of aluminium refining has attracted much research in recent years^31^. Most of the work is focused on understanding the phenomena occurring during the process, which is possible through the use of physical modelling^32^. Increasingly, this modelling is supplemented and at the same time verified by numerical modelling^33^. However, much research is still based on experiments conducted directly in industrial settings^34^. Such testing for the refining of aluminium alloys is mainly carried out to describe various effects that would otherwise be difficult to detect^35^. An example of this is the testing of different types of rotor materials^36^. During refining, the rotors are in direct contact with the molten material, which significantly affects their service life^37^. In order to obtain appropriate results, it is therefore necessary to conduct experiments directly in the operating conditions of the foundry^38^. The study of the efficiency of degassing depending on the wear of the rotor^39^, and the study of the operation of various types of refining gases (Ar, N_2_) should also be mentioned.

The purpose of the article was to determine the effectiveness of hydrogen removal from a specific aluminium alloy by blowing small gas bubbles in the FDU device and to develop a research methodology for the correct assessment of the effectiveness of the refining process, which can be used in the case of various rotors (of different design) and with various operational parameters, such as the refining gas intensity, rotor speed, or length of the recycling cycle. The assessment will focus on determining the efficiency of refining on the basis of the degree of gasification of the melt, the Dichte Index, and the parameters will be compared with the values obtained from the model data.

## Materials and experimental methods

**Figure 1:**
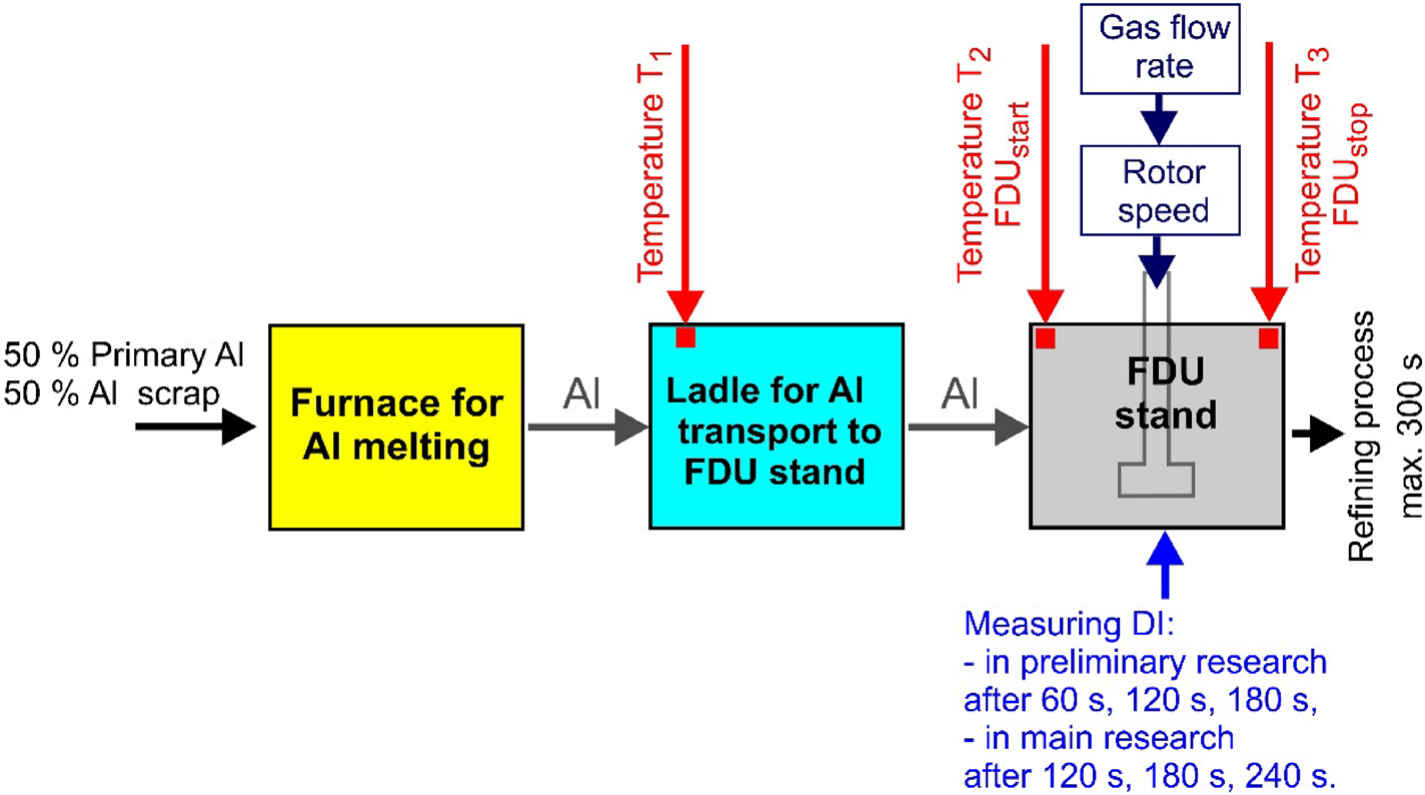
Diagram of the test stand marked with temperature measurements at various stages of the refining process.

Figure 1 shows a diagram of the test stand. MOTOR JIKOV Slévárna a.s. (Die-casting Division) produces castings from AlSi9Cu3(Fe) alloys using high-pressure technology. The aluminium alloy for refining is prepared in the STRIKO MH II-T 2000/1000 melting furnace. Recycled and new material is fed into the furnace with a maximum of 50% recycled material and a minimum of 50% new material (within 5% error) – it is the most economical case applied in the foundry. The alloy melts at 720 ± 20 °C. The chemical composition of the charge material AlSi9Cu3(Fe) is regulated by DIN 226^40^. As part of checking the chemical composition, a sample of the alloy is taken from each batch of refining material for analysis. Table 1 presents the average chemical composition of samples taken in a given series of experiments, obtained by optical emission spectrometry. The results of the chemical composition analysis corresponded to the assumed chemical composition and showed that the tested alloy was not contaminated with other elements.

**Table 1:** The average chemical composition of the AlSi9Cu3(Fe) alloy samples taken during the test versus chemical composition according to DIN 226^40^.

	Chemical composition (wt.%)											
	Si	Fe	Cu	Mn	Mg	Cr	Ni	Zn	Pb	Sn	Ti	Al
DIN 226	8–11	1.3 max	2–4	0.55	0.05–0.55	0.15 max	0.55 max	1.2 max	0.35 max	0.25 max	0.25 max	residue
Analysed alloy	9.075	0.793	2.075	0.260	0.244	0.038	0.091	0.900	0.054	0.022	0.045	86.354

The melt prepared in this way is then transported with a Pyrotek 76375E ladle to the refining station. The ladle must be preheated before the metal is poured into it to minimize heat loss during smelting and reduce the risk of damage to the lining. Liquid metal is poured into about 1/3 of the ladle, then an appropriate amount of refining salt is poured onto the melting surface and the crucible is filled with additional metal up to a mass of about 350 kg. In order to estimate the temperature drop, the temperature in the ladle was measured each time and marked as (T_1_). The ladle is then transported to the FDU Roto-Stativ 1–5201 refining station, where the refining process takes place. Here, in addition to the refining unit, there is also a control panel and pressure cylinders with refining gas (nitrogen). FDUs are programmed to change optional process parameters such as gas flow rate, rotor speed, and rotor height from the bottom of the ladle. These parameters are mostly regulated by the manufacturer or supplier of the graphite elements. The general range of technological parameters used at MOTOR JIKOV Slévárna a.s. are given in Table 2.

**Table 2:** Range of technological parameters used at MOTOR JIKOV Slévárna a.s.

Technological parameters	
Degassing time	Purity grade I – 3 min
	Purity grade II – 6 min
	Purity grade III – 9 min
Rotor speed during work cycle (degassing)	300–350 rpm
Rotor speed during rinse cycle (drive to working position)	50 rpm
Nitrogen flow rate during the work cycle	15–17 Nl·min^−1^
Nitrogen flow rate during the rinse cycle	5–7 Nl·min^−1^

The aim of the experiments was to verify the technological parameters of the rotors, designed on the basis of physical modelling and the operational experience of technologists from MOTOR JIKOV Slévárna a.s. (Die-casting division). As part of physical modelling^22^, a number of variants of technological parameters were proposed and tested, which fell within a relatively wide range. Due to the time-consuming nature of conducting operational tests, only those variants whose application in operational conditions is realistic were selected. As part of the research, the course of refining per unit of time was carried out in order to assess changes in refining efficiency during the refining cycle at the FDU. The focus was mainly on determining the moment of the greatest decrease in hydrogen content in the melt and whether it would be possible to shorten the length of the refining cycle, which would be important from an economic point of view. As part of the research, the temperature drop in various aggregates on the way from the melting furnace to the refining station was also examined.

Experimental research was divided into two stages: preliminary research and main research. Preliminary tests were carried out using the A rotor. Before starting the main experiments, a test batch of melts ("0") was collected. The aim of this series was to develop a methodology for operational tests and a way to evaluate the results. The operational parameters that did not change during the tests were specified. These parameters corresponded to the standard setting, commonly used in the operating conditions of a given type of rotor. The operating parameters for the tests are listed in Table 3.

**Table 3:** Technological parameters of the refining process at the FDU – preliminary research (rotor A).

Rotor	Refining process parameters		Rotor view
A	Degassing time → refining	60 to 180 s	
	Rotor speed during the work cycle (degassing)	330 rpm	
	Flow rate of refining gas (nitrogen/argon) during the work cycle	17.5 l∙min^−1^	
	Position of the rotor during the work cycle (distance from the bottom)	200 mm	

**Figure 2:**
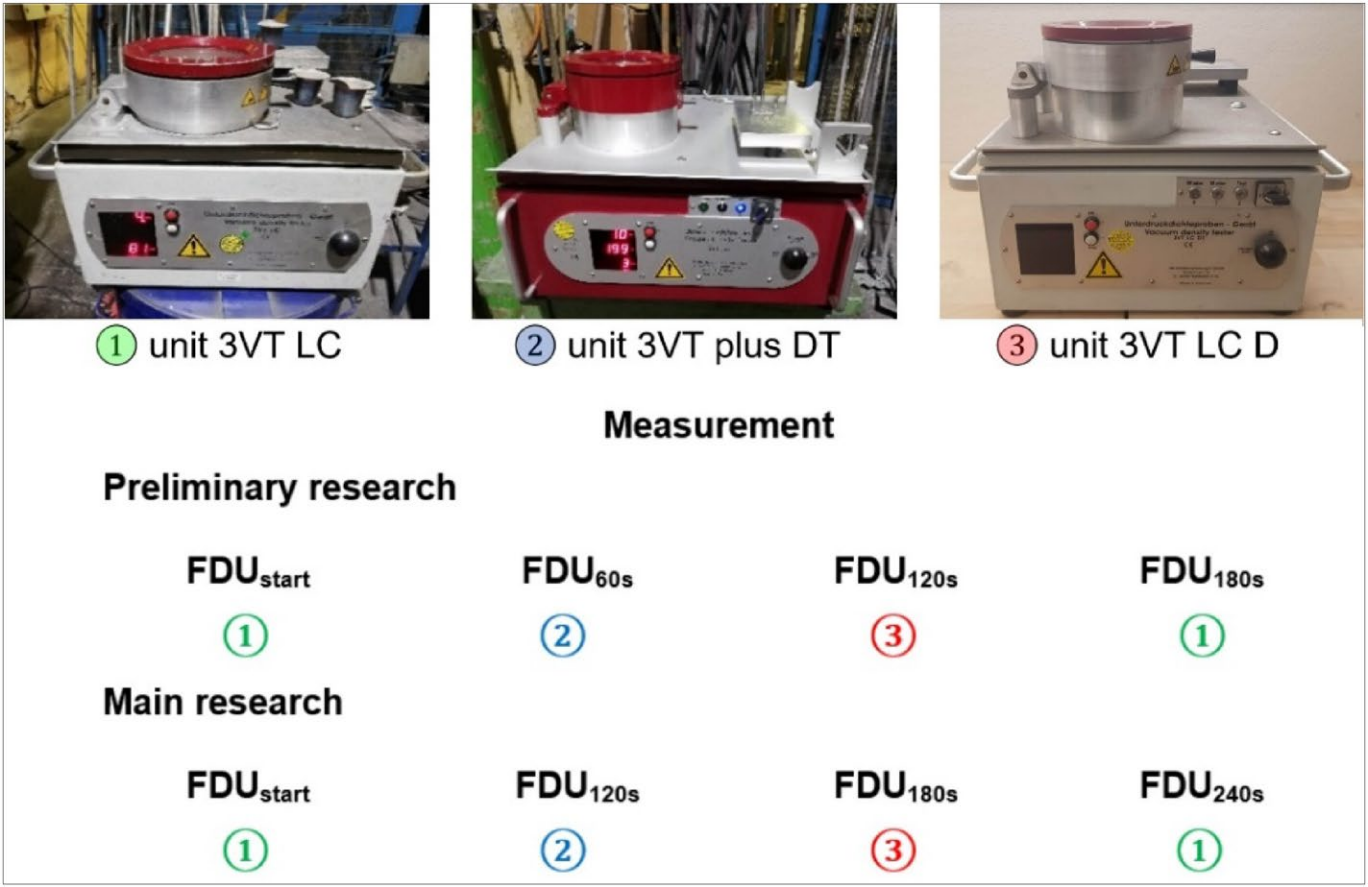
Scheme of Dichte Index measurement during the preliminary and main tests.

As part of the operational experiments, samples were taken to assess the degassing of the aluminium alloy. For this purpose, devices from mk AL-SCHMELZE-MESSTECHNIK were used for vacuum testing of the density index (Dichte Index DI – a description of the methodology is presented in the article^18^), which is presented in Fig. 2. To simultaneously measure the Dichte Index for samples taken during one refining cycle (4 samples at four different times—for preliminary tests after 0 s, 60 s, 120 s, 180 s and for main tests after 0 s, 120 s, 180 s and 240 s) three devices were used: 3VT LC (allows determination of Dichte Index only), 3VT LC DT (allows also performing Drosstest (DT) in addition to Dichte Index), 3VT plus DT device (multifunctional device, which allows determination of Dichte Index (DI), Drosstest (DT) and Straube-Pfeiffer test (SPT))—the measurement scheme is also shown in Fig. 2. After successfully testing the measurement methodology and evaluating the results, the main research was carried out. As part of these experiments, various combinations of operating parameters were tested, designed on the basis of a physical model and the operational experience of technologists from MOTOR JIKOV Slévárna a.s. The design of the variants was based on the standard conditions for dispersing gas bubbles for a given rotor, which are summarized in Table 4.

**Table 4:** Technological parameters of the refining cycle carried out in the industry as part of the main research.

Rotor	Refining process parameters		Rotor view
B	Material	Graphite	
	Refining time	120 to 240 s	
	Rotary impeller (rotor) speed	300–400 rpm	
	Nitrogen flow rate	15–19 l∙min^−1^	
	Working height	160 mm	

The tests within the main research series were aimed at evaluating the efficiency of refining in the refining cycle at the FDU using the B impeller, which is characterised by good degassing efficiency. It is a pump-type impeller that usually requires high rotational speeds to achieve a uniform distribution of gas bubbles throughout the volume of the metal. In addition to rotary impeller speed and refining gas flow rate, the influence of the refining cycle length on the degassing efficiency in the range from 120 to 240 s was also investigated. For the purpose of these experiments, the experimental methodology was set up similarly to the preliminary studies ("0"), with sampling intervals adjusted to the length of the cycle. Samples were taken before and after refining and after 120 and 180 s of the process, i.e. in the intervals FDUstart, FDU120s, FDU180s and FDU240s. It should also be added here that the tests presented in the article were carried out during normal operation of the foundry. Therefore, the test series of melts ("0") differs in the rotor used type A, which was attached to the FDU station during the experiments. However, this is not a problem from the point of view of the established methodology of further experiments. In addition, both types of rotors are produced by the same manufacturer.

In total, 27 variants of experiments were defined, the details of which are presented in Table 5. These variants differed in rotary impeller speed and refining gas flow rate.

**Table 5:** Specification of operational experiment variants – main research.

Series of experiments	Variant	Rotary impeller (rotor) speed (rmp)	Flow rate of refining gas during the work cycle (l·min^−1^)	Time cycle (s)
I	1	300	15	120
	2			180
	3			240
II	4		17	120
	5			180
	6			240
III	7		19	120
	8			180
	9			240
IV	10	350	15	120
	11			180
	12			240
V	13		17	120
	14			180
	15			240
VI	16		19	120
	17			180
	18			240
VII	19	400	15	120
	20			180
	21			240
VIII	22		17	120
	23			180
	24			240
IX	25		19	120
	26			180
	27			240

Thanks to the properly selected sampling methodology, it was possible to obtain data for 3 defined variants, differing in cycle length, during the measurement of one series of experiments with constant speed and flow; e.g. data for variants 1, 2 and 3 were obtained during the measurement of the refining cycle at the rotary impeller speed of 300 rpm, flow rate of 15 Nl min^−1^ and time of 240 s. Thus, the total series of experiments was reduced to 9, in which data for 27 defined variants were measured, which meant a significant time saving (1 series of measurements = 1 day).

## Results of the research

**Figure 3:**
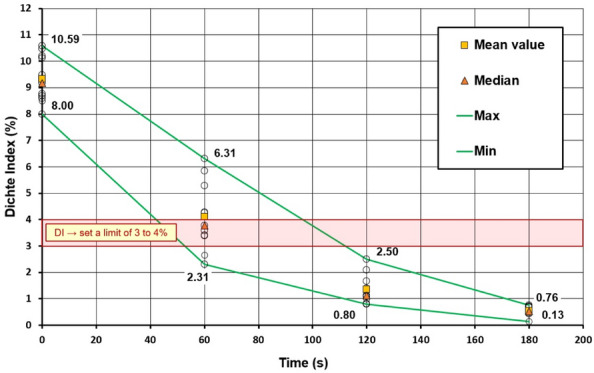
Degassing curve – change of Dichte Index values during the refining cycle in the preliminary test.

Figure 3 shows the results of the refining tests obtained as part of the preliminary tests. A degassing curve was constructed based on the measured Dichte Index values. In the graph, the limits of the maximum and minimum measured Dichte Index values along with the mean value and the median are marked in green. To ensure the required quality of castings, the maximum allowable Dichte Index value ranged from 3 to 4% (range marked in red). Based on the results obtained from the industrial experiments, it was noticed that the dispersion of Dichte Index values decreases with the decrease in the hydrogen content in the melt. During the refining cycle, approximately 93% of the hydrogen contained in the alloy was removed, with the greatest decrease in the hydrogen content visible in the first 120 s of the process, when the average Dichte Index value dropped to 1.42%, so practically 80% of the hydrogen contained in the alloy has been removed after this time, which also means that the required hydrogen content limit (DImax = 3 to 4%) has been reached after this time. On this basis, it can be concluded that under certain technological conditions and using the A rotor, it is possible to shorten the refining cycle from 180 to 120 s while maintaining the required metal quality.

**Figure 4:**
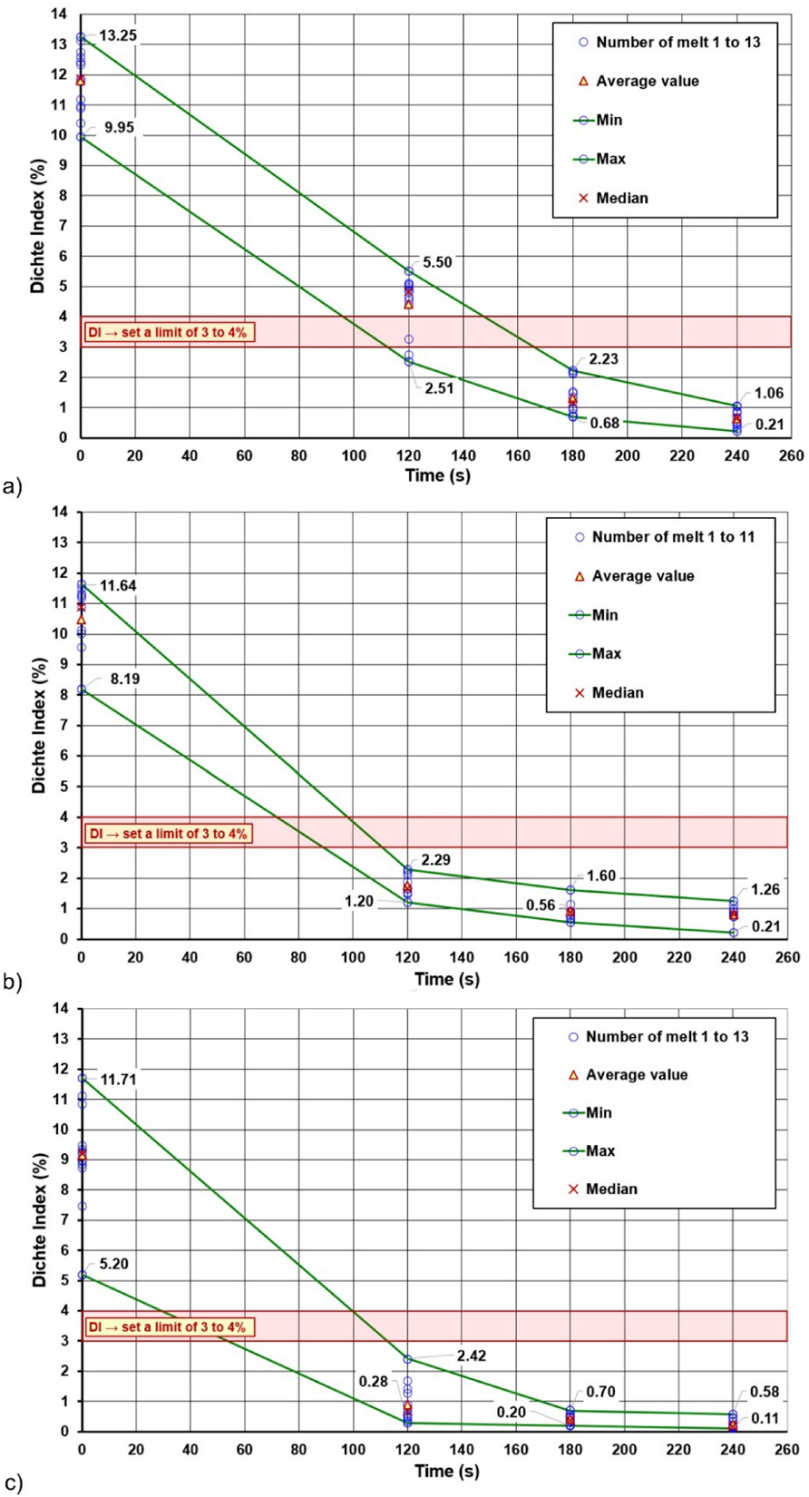
Degassing curves plotted on the basis of the measured Dichte Index values for a gas flow rate of 17 l/min and the following rotary impeller speed: (**a**) 300 rpm (variant II), (**b**) 350 rpm (variant V), (**c**) 400 rpm (variant VIII).

**Figure 5:**
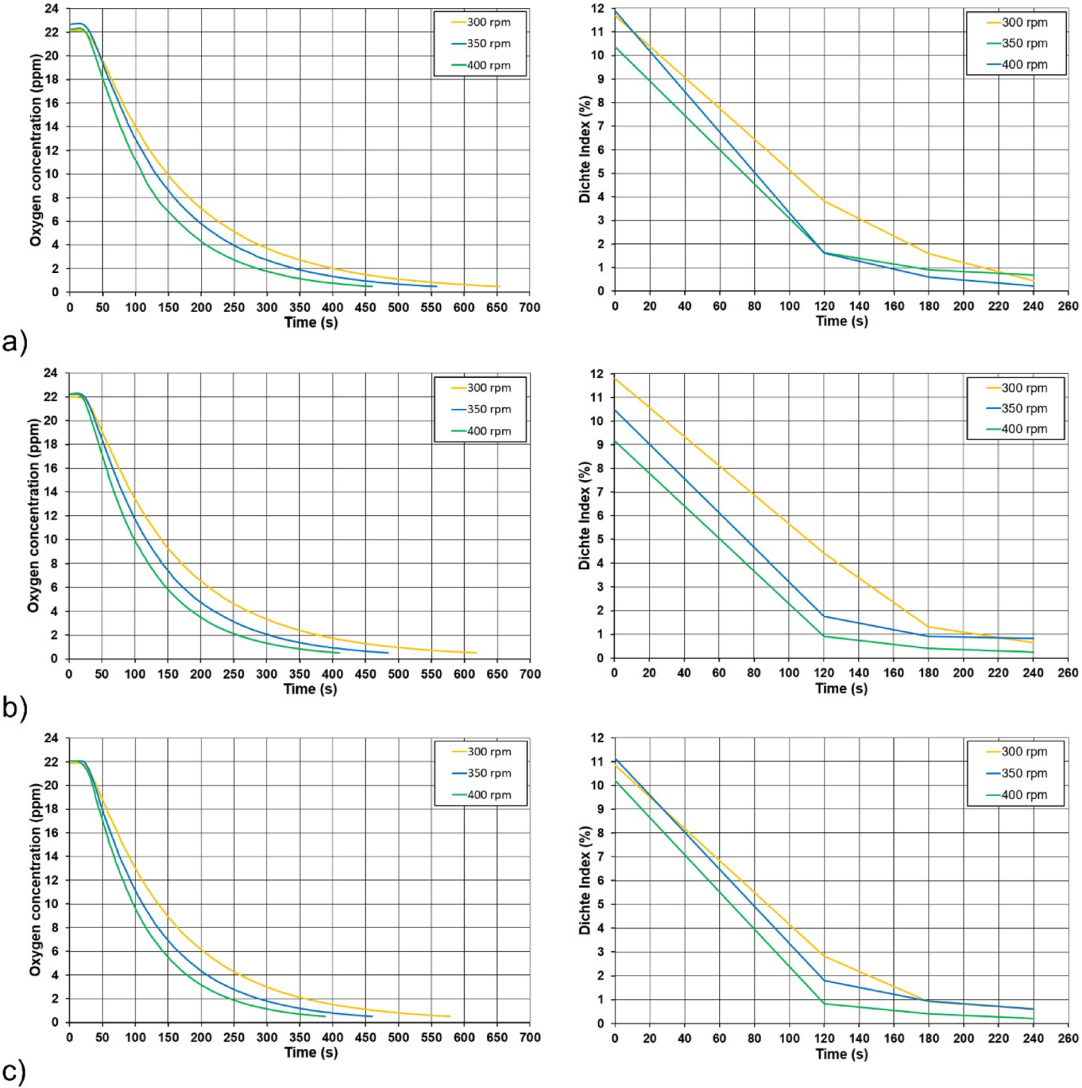
Comparison of the degassing curves (removal of oxygen/hydrogen from the model fluid/aluminium alloy) obtained from model tests (left) with the results obtained in industrial conditions (right) for different rotary impeller speeds for gas flow rates: (**a**) 15 l/min, (**b**) 17 l/min, (**c**) 19 l/min.

In the case of the main tests, the procedure was similar to the preliminary tests—on the basis of the measured data for each series of experiments, degassing curves were constructed which express the decrease in the hydrogen content in the alloy by the Dichte Index value over time. Figure 4 shows the degassing curves for series (II), (V) and (VIII), which show the effect of the rotary impeller speed on the degassing process under operating conditions with a constant refining gas flow rate of 17 l/min. On the presented curves, a downward trend of the Dichte Index value can be observed with increasing rotary impeller speed. This may, to some extent, confirm the results of measurements on the physical model, where the removal of oxygen from water was increased with the increase of the rotary impeller speed^21^. In the case of data obtained from industrial research, the trends were not as clear as in the case of the results obtained on the physical model—such a comparison is presented in Fig. 5. Despite different values on the y-axis, in the case of physical modelling, oxygen concentration was measured in ppm, and in the case of the industrial research, the Dichte Index (hydrogen removal rate) was determined in %, similarities in the obtained results can be seen. This confirms the usefulness of using physical modelling to determine the process parameters of the rotors. However, some discrepancy in the results is understandable, considering that physical modelling was carried out in laboratory conditions, where there are fewer variables and it is easier to ensure the same conditions for each experiment. One should also take into account the increasing wear of the rotor, which is already known to have a positive effect on its efficiency^39^. In this case, however, the effect of wear cannot be taken into account, because the tests were carried out with the most worn rotor of the variant with the lowest rotary impeller speed, which, however, turned out to be the least efficient. If rotor wear affected the measured Dichte Index values, it did not affect the observed trend in any way.

**Figure 6:**
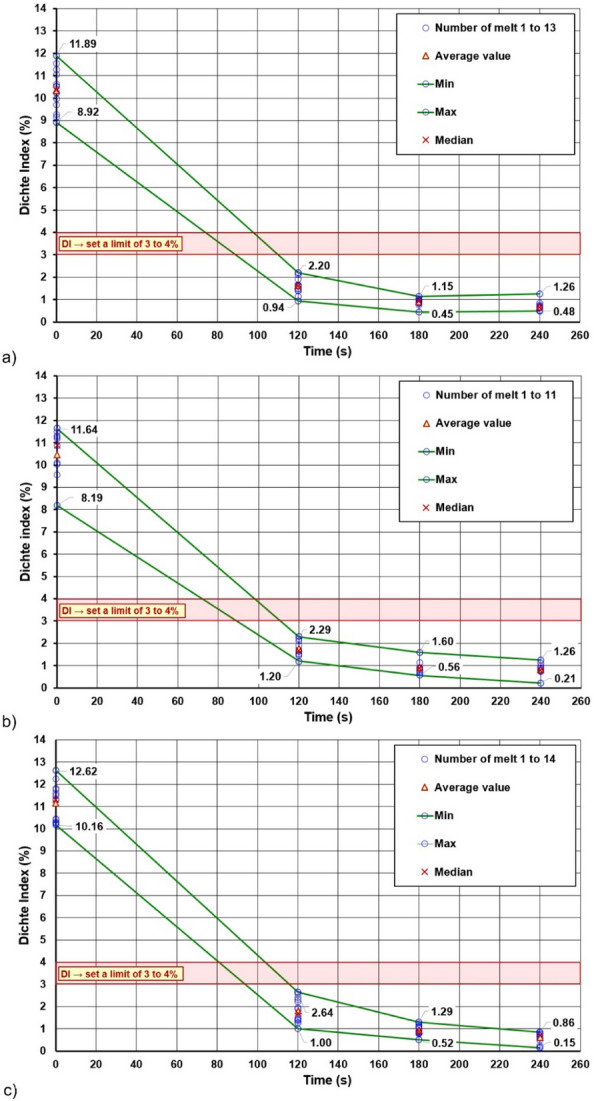
Degassing curves developed on the basis of the measured Dichte Index values for the rotor speed of 350 rpm and different gas flow rates: (**a**) 15 l/min (variant IV), (**b**) 17 l/min (variant V), (**c**) 19 l/min (variant VI).

Figure 6 shows the degassing curves for experiment series (IV), (V) and (VI), showing the effect of refining the gas flow rate. Based on these results, it can be concluded that the effect of the gas flow rate on the process in industrial conditions is ambiguous. The curves are very similar in nature and it cannot be said that the refining efficiency increased with increasing gas flow rate. Also in this case, the impact of wear should not be significant, because the rotors used for the tests in a given series showed a similar number of cycles.

**Figure 7:**
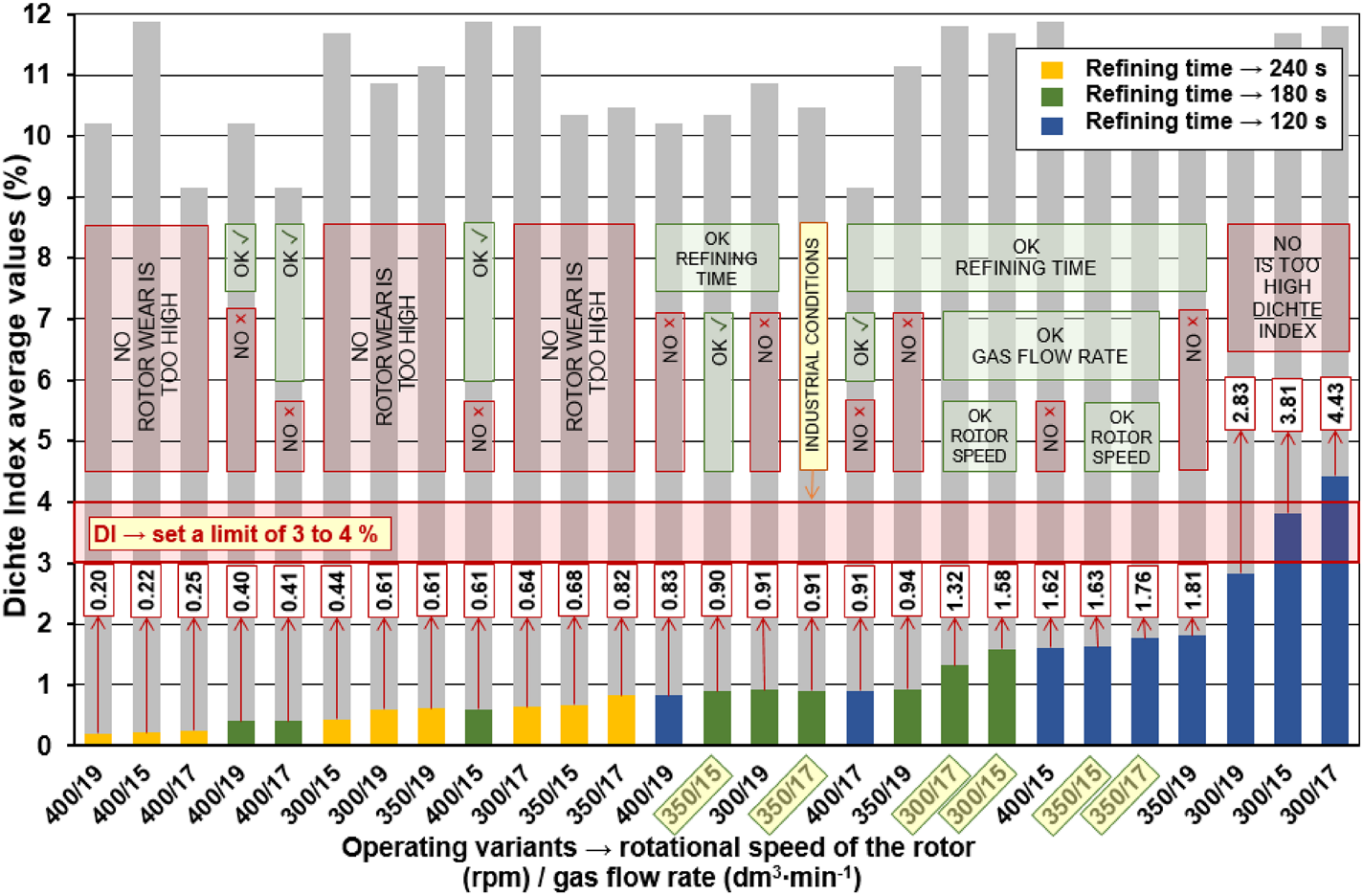
Cumulative test results to assess the efficiency of the refining process for the selected B rotor at the FDU station.

Figure 7 shows the cumulative assessment of the efficiency of the liquid aluminium alloy refining process carried out in the FDU, based on the results measured in series (I) to (IX). On the basis of this diagram, variants suitable for the operation of the tested rotor in industrial conditions were indicated. The goal of designing optimised refining conditions, i.e. for achieving financial savings while maintaining or increasing the efficiency of the process, was taken into account. In addition, it was also taken into account that (a) the average values of the measured Dichte Index had to reach a maximum of 3 to 4%, (b) at rotary impeller speeds of 400 rpm graphite elements were degraded faster, and (c) that from the point of view of process economics, it is not advantageous to work at a gas flow rate of 19 l/min, because such a high flow rate does not bring a corresponding increase in the efficiency of the refining process.

In Fig. 7, the variants recommended for use in industrial foundry conditions are marked in green on the x-axis. These are variants with the following combinations of technological parameters:

- Variant 11 → speed: 350 rpm, gas flow rate: 15 l/min, refining time: 180 s,
- Variant 14 → speed: 350 rpm, gas flow rate: 17 l/min, refining time: 180 s,
- Variant 5 → speed: 300 rpm, gas flow rate: 17 l/min, refining time: 180 s,
- Variant 2 → speed: 300 rpm, gas flow rate: 15 l/min, refining time: 180 s,
- Variant 10 → speed: 350 rpm, gas flow rate: 15 l/min, refining time: 120 s,
- Variant 13 → speed: 350 rpm, gas flow rate: 17 l/min, refining time: 120 s.

With the above assumptions, the remaining variants turned out to be insufficient to improve the economics of the process. The variants with a rotary impeller speed of 300 rpm and a cycle length of 120 s after degassing exceeded the specified maximum limit of the Dichte Index. Variants with a rotary impeller speed of 400 rpm and a cycle length of 240 s were rejected due to the rapid wear of the graphite elements. Variants with a gas flow rate of 19 l/min are not economically advantageous. In addition, variants 11 and 14 with rotary impeller speeds of 350 rpm show very similar final values of the Dichte Index. The same can be said for variants 2, 5, 10 and 13. Those variants achieved slightly higher Dichte Index values compared to variants 11 and 14. However, the use of variants 2, 5, 10 and 14 could bring financial savings in operating conditions due to lower rotary impeller speeds or shorter refining time.

For these variants, samples were taken and the Dichte Index measured to confirm the appropriate degree of degassing of the alloy; the results are shown in Table 6. The presented results confirm the above selection of variants. This is particularly visible in the case of variant 2 (speed: 300 rpm, gas flow rate: 15 l/min, refining time: 180 s), where the presented sample after degassing is characterized by a lack of porosity. In the same row of Table 6 there is also variant 1 (speed: 300 rpm, gas flow rate: 15 l/min, refining time: 120 s). Variant 1 differs from variant 2 in degassing time, in the case of variant 1 this time is 120 s – which is too short to remove the appropriate amount of hydrogen so that its compactness does not cause porosity, which can be seen on both sides of the sample cross-section. In the case of variants 10 and 11 as well as 13 and 14, it can be seen that both cross-sections of the samples are clean after 120 s and 180 s, without porosity. Therefore, if after 120 s the level of hydrogen removal obtained is sufficient, it can be concluded that 120 s in this case will be more profitable from the economic point of view.

**Table 6:**
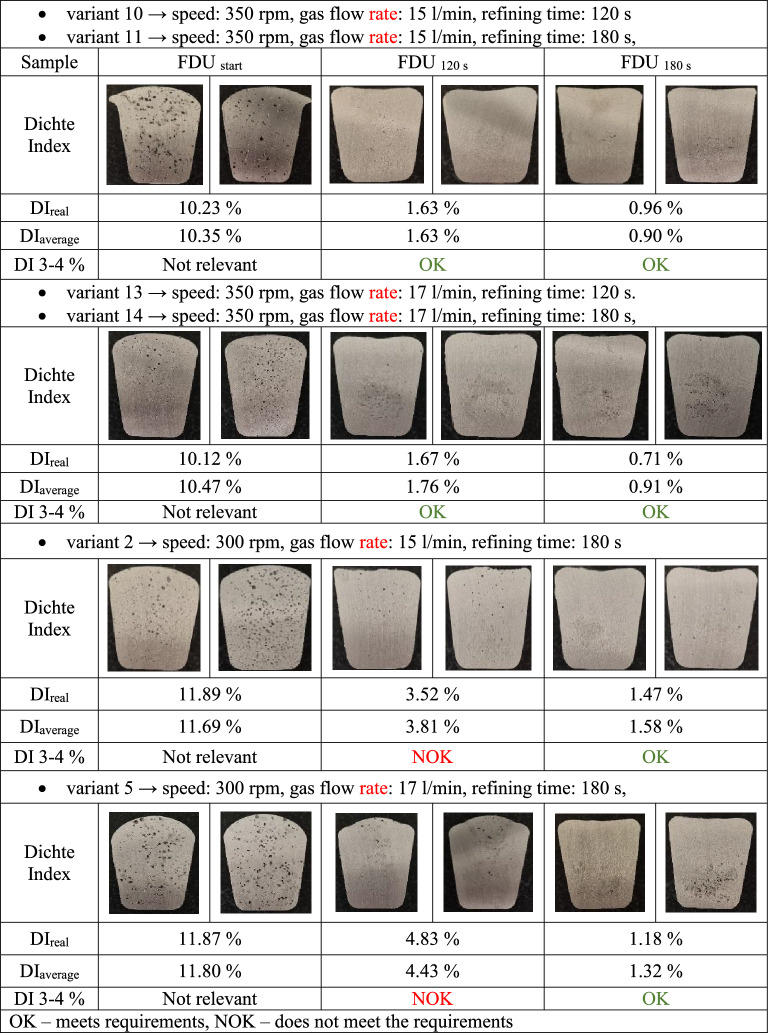
Results of Dichte Index measurements – section through the casting and view of the degree of degassing with the corresponding DI values.

**Figure 8:**
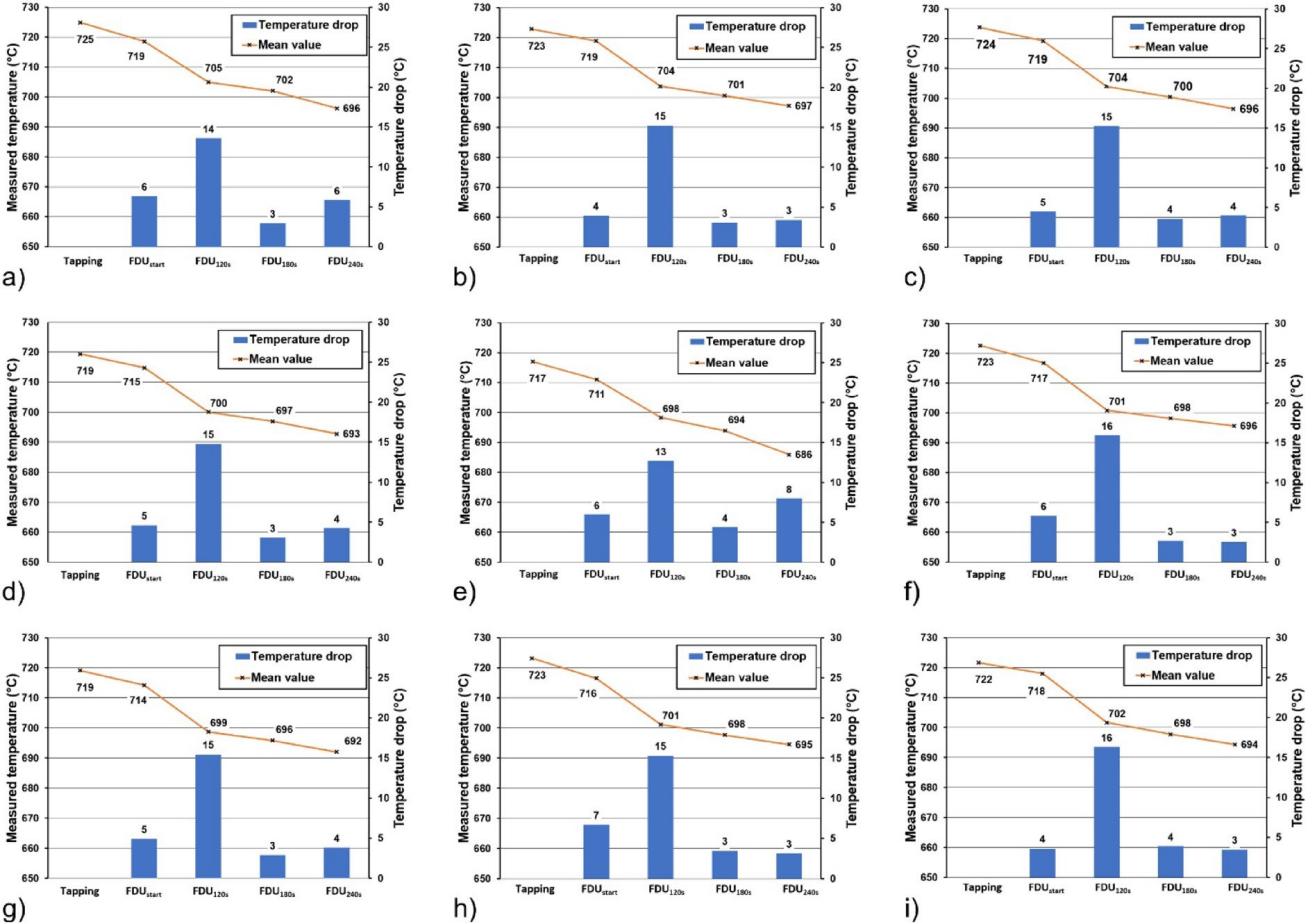
Process temperature changes from the moment of tapping the molten alloy from the melting furnace to the transport ladle to the end of the refining process (FDUstop) for each series of experiments: (**a**) I, (**b**) II, (**c**) III, (**d**) IV, (**e**) V, (**f**) VI, (**g**) VII, (**h**) VIII, (**i**) IX.

In addition, part of the experiments in industrial conditions comprised the measurement of the temperature during the refining cycle for all the tested variants. The results are summarized in Fig. 8. The temperature was measured at different intervals of the cycle time. The first moment of the measurement was tapping the melt into the transport ladle or refining reactor, and then taking samples for the Dichte Index assessment, i.e. in the Tapping, FDUstart, FDU120s, FDU180s and FDU240s ranges.

The curves plotted in Fig. 8 show the decrease in temperature during the refining cycle. For better visualization, the temperature losses were calculated from the measured values, which express the temperature drop from the tapping ladle to the set measurement range. It can be seen that between the time the melt is tapped into the transfer ladle and the start of the refining cycle (FDUstart) the melt has lost about 4 to 7 °C. The greatest decrease in temperature occurred in the first 120 s of the refining process (FDU120s), when a temperature loss of 19 to 22 °C was observed. During the further refining process up to 240 s (from FDU180s to FDU240s), the temperature drop was not so drastic. The total temperature drop from pouring the molten material into the ladle to completion of refining was 26 to 32 °C, which is within the limits acceptable to the foundry.

## Summary

The tests carried out and the results obtained allow the following conclusions to be drawn:

- Modelling research allows finding out more about the phenomena occurring during the refining process of aluminium and its alloys by blowing with refining gases and to determine the optimal processing parameters such as gas flow rate or rotary impeller speed. Such tests, however, must be verified in industrial conditions. The results obtained during physical modelling and in industrial conditions are similar, but some discrepancies may result from the uncontrolled course of research in industrial conditions.
- In the case of industrial research, it is very important to define the research procedure. This allows the testing of different types of rotors and their impacts on the process efficiency, and at the same time proper evaluation of the results of the hydrogen removal process from liquid aluminium and its alloys.
- In order to select the optimal conditions for the refining process in industrial conditions, certain assumptions were made resulting from industrial experience and the economics of the process: the average values of the measured Dichte Index had to be between 3 and 4%; the values of the rotary impeller speed above 400 rpm were rejected due to the accelerated degradation of the graphite elements of the rotor; the values of the refining gas flow rate above 19 l/min were also rejected for economic reasons and the fact that such a high flow rate does not ensure an adequate increase in the efficiency of the refining process. It was also taken into account that extending the refining time also accelerates the degradation of the graphite elements of the rotor.
- Analysis of the results of the tested variants allowed determination of the six best variants of the refining process in terms of rotary impeller speed (300 or 350 rpm), gas flow rate (15 or 17 l/min) and refining time, which in the best case should be 120 s, or 180 s with lower processing parameters. The Dichte Index was measured for these variants, which allows the determination of the level of porosity across the cross-section of the sample, and which confirmed the correctness of the choice of the six indicated variants 2, 5, 10, 11, 13 and 14 as the most optimal.
- During the process, the temperature drop from the moment of transporting the liquid metal from the melting furnace to the refining ladle to the end of the refining process was also measured. The greatest decrease in temperature occurred in the first 120 s of the refining process at the level of 19 to 22 °C. During further refining (240 s) no major temperature changes were observed. The total temperature drop from pouring the molten material into the ladle to the end of refining, which ranged from 26 to 32 °C, was within the limits allowed by the foundry and did not cause major disturbances to the plant operation.
- The experiments enabled a research methodology to be developed to increase the efficiency of the refining process, suitable for use in operating conditions. However, there are still many opportunities to continue this research, whether by changing the shape of the rotor or the material from which it is made. Also, the results of inappropriate combinations of rotary impeller speed and gas flow rate were used to check the wear rate of the rotor, and then its effect on degassing in the condition of poor quality wear.

## Data Availability

The datasets used and/or analysed during the current study available from the corresponding author on reasonable request.
